# Mental Health Services Utilization among Suicidal Patients: Comparing the Impact of Co-Occurring Opioid or Other Substance Use Disorders

**DOI:** 10.1192/j.eurpsy.2023.775

**Published:** 2023-07-19

**Authors:** J. Pathak

**Affiliations:** Population Health Sciences, Cornell University, New York, United States

## Abstract

**Introduction:**

Prior literature establishes bidirectional associations between suicide and substance use disorders (SUDs), particularly opioid use disorder (OUD). However, the context of mental health services utilization remains under-investigated. This analysis examined patterns of mental health services utilization in patients with SUDs and suicidality, identified associated risk factors, and evaluated the impact of patient engagement on subsequent mental health outcomes

**Objectives:**

See above.

**Methods:**

Electronic health records (EHRs) derived from 7 health systems across New York City between 2010-2019 were analyzed. Suicidality was identified as any ICD-9/10 diagnosis of suicide attempt, suicidal ideation, or self-harm injury. SUDs were identified as any opioid, cannabis, cocaine, hallucinogen, inhalant, sedative/hypnotic/anxiolytic, amphetamine, or other substance abuse or dependence. Quasi-Poisson regression adjusted for age, gender, and chronic diseases was used to model associations between OUD exposure and the frequency of encounters and estimate the relative risk (RR) of significant covariates.

**Results:**

A total of 6977 adults with suicidality and any comorbid SUD were selected, including 2203 (31.6%) with a diagnosis of OUD and 4774 (68.4%) without a diagnosis of OUD. Most patients were male (54.8%) and aged between 25-64 years (79.3%). Many (61.3%) had over 3 chronic diseases, including depression (80.8%), hypertension (60.6%), anemia (43.0%), and hyperlipidemia (41.9%). Compared to patients with other SUDs, those with OUD had higher odds of self-harm injury [OR: 1.26 (95% CI: 1.13-1.41)], depressive disorders [1.47 (1.29-1.67)], anxiety disorders [1.65 (1.48-1.84)], psychotic disorders [1.23 (1.11-1.37)], personality disorders [1.30 (1.16-1.48)], and post-traumatic stress disorder [1.37 (1.20-1.57)]. Patients with OUD were more likely to utilize all-cause outpatient (RR: 1.16), emergency department (ED) (RR: 1.43), and inpatient (RR: 1.60) services (p<0.001). Among OUD patients, males were less likely to have outpatient visits (RR: 0.79) and inpatient hospitalizations (RR: 0.88), and older age was protective against ED admissions (RR range: 0.62-0.71). Additionally, individuals with OUD were more likely than those with other SUDs to have SUD-related encounters, as well as suicide-related ED admissions and inpatient hospitalizations (p<0.0001). Those who had more mental health outpatient visits were less likely to have suicide-related ED admissions (RR: 0.85), however this association was weaker among younger or male patients with comorbid OUD.

**Conclusions:**

Among suicidal adults with comorbid SUDs, those with a diagnosis of OUD were more likely to utilize mental health services and have psychiatric comorbidity. Males and older adults were less likely to utilize services.

**Disclosure of Interest:**

None Declared

